# The 2.1 Å Resolution Structure of Cyanopindolol-Bound β_1_-Adrenoceptor Identifies an Intramembrane Na^+^ Ion that Stabilises the Ligand-Free Receptor

**DOI:** 10.1371/journal.pone.0092727

**Published:** 2014-03-24

**Authors:** Jennifer L. Miller-Gallacher, Rony Nehmé, Tony Warne, Patricia C. Edwards, Gebhard F. X. Schertler, Andrew G. W. Leslie, Christopher G. Tate

**Affiliations:** Structural Studies Division, MRC Laboratory of Molecular Biology, Cambridge, Cambridgeshire, United Kingdom; University of Cambridge, United Kingdom

## Abstract

The β_1_-adrenoceptor (β_1_AR) is a G protein-coupled receptor (GPCR) that is activated by the endogenous agonists adrenaline and noradrenaline. We have determined the structure of an ultra-thermostable β_1_AR mutant bound to the weak partial agonist cyanopindolol to 2.1 Å resolution. High-quality crystals (100 μm plates) were grown in lipidic cubic phase without the assistance of a T4 lysozyme or BRIL fusion in cytoplasmic loop 3, which is commonly employed for GPCR crystallisation. An intramembrane Na^+^ ion was identified co-ordinated to Asp87^2.50^, Ser128^3.39^ and 3 water molecules, which is part of a more extensive network of water molecules in a cavity formed between transmembrane helices 1, 2, 3, 6 and 7. Remarkably, this water network and Na^+^ ion is highly conserved between β_1_AR and the adenosine A_2A_ receptor (rmsd of 0.3 Å), despite an overall rmsd of 2.4 Å for all Cα atoms and only 23% amino acid identity in the transmembrane regions. The affinity of agonist binding and nanobody Nb80 binding to β_1_AR is unaffected by Na^+^ ions, but the stability of the receptor is decreased by 7.5°C in the absence of Na^+^. Mutation of amino acid side chains that are involved in the co-ordination of either Na^+^ or water molecules in the network decreases the stability of β_1_AR by 5–10°C. The data suggest that the intramembrane Na^+^ and associated water network stabilise the ligand-free state of β_1_AR, but still permits the receptor to form the activated state which involves the collapse of the Na^+^ binding pocket on agonist binding.

## Introduction

There are three β-adrenoceptors (βARs) encoded by the human genome, β_1_AR, β_2_AR and β_3_AR, which are all members of the G protein-coupled receptor (GPCR) superfamily [1], [2]. The development of novel engineering strategies for GPCRs [3] has allowed the structures of both β_1_AR and β_2_AR to be determined bound to a variety of agonists, partial agonists and inverse agonists [4]–[12]. In addition, the structure of β_2_AR has been determined in complex with a heterotrimeric G protein [7]. Receptor activation is characterised by a large outward movement of the cytoplasmic ends of transmembrane helices H5 and H6, which opens up a cleft at the cytoplasmic face of the receptor, allowing G protein binding and, hence, activation of the G protein.

The structures determined to date show how ligands of different classes bind to the receptor and give an insight into the reasons of their respective efficacies [13]. Structures of β_1_AR bound to antagonists represent the inactive R state of the receptor, with Ser212^5.43^ (Ballesteros-Weinstein nomenclature [14] in superscript) making an intrahelical hydrogen bond and Ser215^5.46^ forming a hydrogen bond with the side chain of Thr126^3.37^ (refs [5], [11]). Binding of a full agonist causes a contraction of the ligand binding pocket by ∼1 Å and the rotamer conformational changes of Ser215^5.46^ and Ser212^5.43^, which combine to weaken the helix-helix interactions between H3-H4-H5 [10]. In contrast, binding of partial agonists stabilises the contraction of the ligand binding pocket and the rotamer conformation change of Ser212^5.43^, but not a rotamer change of Ser215^5.46^ (Ref [10]). Inverse agonists block the rotamer conformational change of Ser215^5.46^ (Refs [5], [10]). However, many questions still remain about both the dynamics of these processes and about the structures themselves, which may be answered through higher resolution structures of different states of the receptors produced in lipidic cubic phase (LCP).

Previously, crystallisation of GPCRs in LCP [15], [16] has depended on making GPCR chimeras [17] with T4 lysozyme (T4L) or BRIL inserted into cytoplasmic loop 3 (CL3), which has led to the structure determination of many different GPCRs [2]. However, on occasion features on the cytoplasmic face of the receptor may be perturbed by the T4L, such as the conformation of CL2 in β_2_AR (discussed in ref [11]) and the unusual orientation of the cytoplasmic ends of H5 and H6 in the structure of the adenosine A_2A_ receptor (discussed in ref [18]). The role of T4L is to make crystal contacts, but in theory there should be sufficient hydrophilic surfaces on a native receptor for crystallisation, provided that the protein is sufficiently stable in LCP. Indeed, many small membrane proteins have been crystallised in LCP [19], and these proteins are generally characterised by being stable in detergent solutions. The thermostabilised receptors that we developed for the crystallisation of GPCRs [20]-[25] were therefore more likely to succeed than the wild type receptor. In addition, they were all crystallized previously in detergent solution without the aid of T4L or BRIL fusion proteins. Here we present the 2.1 Å resolution structure of thermostabilised β_1_AR crystallised in LCP without the use of a fusion protein, which has facilitated the identification of an intramembrane Na^+^ ion important in maintaining receptor stability.

## Methods

### Expression, purification and crystallization

The turkey (*Meleagris gallopavo*) β_1_AR construct that was crystallised, β_1_AR-JM50, contained nine thermostabilising point mutations and truncations at the N terminus, inner loop 3, and C terminus [26]. It is identical to the β44-m23 construct previously crystallized [12] apart from the inclusion of three additional thermostabilising mutations, I129V, D322K and Y343L [25]. Receptors were expressed in insect cells and purified bound to (s)-cyanopindolol as described previously [26] and concentrated to 35 mg/mL in 10 mM Tris-HCl, pH 7.6, 100 mM NaCl, 0.1 mM EDTA, 0.1% decylmaltoside. Before crystallisation, receptor was diluted to 25 mg/ml by addition of cholesteryl hemisuccinate to 3 mg/mL from a stock solution in 2% Hega-10 so that the final detergent concentrations were 0.07% decylmaltoside and 0.6% Hega-10. LCP crystallisation set-ups [16] contained a 2∶3 receptor∶monoolein ratio which was dispensed in 100 nl aliquots using a lipid handling instrument designed and built at the MRC Laboratory of Molecular Biology [27], which served as a prototype and inspiration for the mosquito-LCP developed in collaboration with TTP LabTech (Melbourn, UK). Crystals were grown at 22°C with 0.1 M ADA (N-(carbamoylmethyl)imino-diacetic acid) buffer pH 7.0 with PEG 600 (24–28%). Crystals were harvested singly in LithoLoops (Molecular Dimensions Ltd) and cryo-cooled in liquid nitrogen.

### Data Collection, Structure Solution, and Refinement

Diffraction data were collected from a single cryo-cooled crystal (100 K) using a 10 μm focused beam at ID23-2 at a wavelength of 0.8726 Å (ESRF, Grenoble). Fourteen wedges of data were collected from different parts of the crystal, each wedge comprising of 40 images (0.5° rotation per image). Images were processed with MOSFLM [28] and SCALA [29], and finally eight wedges of 20–35 images (10–17.5° rotation) were combined for the final data set (Table 1). The structure was solved by molecular replacement with Phaser [30] using the structure of β_1_AR with cyanopindolol bound (PDB code 2VT4, chain B) as a starting model. There was a single protein chain per asymmetric unit. Refinement, rebuilding, and validation were carried out with REFMAC5 [31], Coot [32], and MolProbity [33], respectively.

**Table 1:** Data processing, refinement and evaluation statistics.

	β44-JM50
Number of crystals	1
Space group	P2_1_22_1_
Unit cell parameters	
a, b, c (Å)	53.1, 62.1, 95.8
α, β, γ (°)	90, 90, 90
**Data Processing**	
Resolution (Å)	31.9–2.1
Rmerge^1^	0.127 (0.540)
<I/σ(I)>^1^	7.8 (1.9)
Number of reflections	71427 (5974)
Unique reflections	18686 (2515)
Completeness (%)^1^	98.0 (92.5)
Multiplicity^1^	3.8 (2.4)
Wilson B factor (Å^2^)	21.9
**Refinement**	
Total number of reflections	17696
Total number of atoms	2506
Number of waters	38
Number of lipid molecules#	7
Number of sodium ions	2
R_work_ 2 ^,^ 4	0.193 (0.249)
R_free_ 3 ^,^ 4	0.245 (0.330)
r.m.s. deviation bonds (Å)	0.146
r.m.s. deviation angles (°)	1.56
Mean atomic B factor (Å^2^)	33.28
Estimated coordinate error (Å)	0.13
Ramachandran plotfavoured (%)*	99.30
Ramachandran plotoutliers (%)*	0

Footnotes.

1. Values in parentheses are for the highest resolution bin (2.21–2.1 Å).

2. Number of reflections used to calculate R_work_ (94.9%).

3. Number of reflections from a randomly selected subset used to calculate R_free_ (5.1%).

4. Values in parentheses are for the highest resolution bin for refinement (2.154–2.1 Å).

#Two lipid molecules were modelled with 50% occupancy due to symmetry issues.

* Figures obtained using MolProbity [33].

### Thermostability assays

Expression of β_1_AR mutants in *E. coli* was performed as described by Serrano-Vega *et al.* [22]. Briefly, XL-10 cell cultures of 500 mL of 2× TY medium containing ampicillin (100 μg/mL) were grown at 37°C until OD_600_ = 3 and then induced with 0.4 mM IPTG. Induced cultures were incubated at 25°C for 4 h, and cells were then harvested by centrifugation. For membrane preparations each cell pellet was resuspended in 15 mL of buffer [20 mM Tris (pH 8), 10 mM MgCl_2_, 10 μg/mL DNase I and protease inhibitors (Complete; Roche)]. The suspension was then sonicated three times for 30 sec and centrifuged for 1 h at 12,000 *g*. The supernatant was ultracentrifuged at 200,000 *g* for 90 min. Finally, the membrane pellet was resuspended with 5 mL of Tris 20 mM (pH 8) supplemented with protease inhibitors, homogenised, aliquoted and flash frozen in liquid nitrogen before storage at −80°C. The total protein concentration was determined by the Bradford method [34]. To check the expression level of each construct, equal amounts of total membrane proteins and 100 nM of ^3^H-DHA were used in the binding assay.

For the thermostability assay for the ligand-free receptor, 1–2.5 mg of *E. coli*-expressed membrane protein was solubilised for 30 min on ice with 1% DDM and insoluble material was removed by centrifugation (5 min, 20,000 *g*). Samples were then adjusted to give the desired concentration of NaCl or choline chloride. Thermostability was measured by incubating the sample at the specified temperature for 30 min; reactions were placed on ice, and ^3^H-DHA was added (50 nM final concentration) and equilibrated (2 h, 4°C). Receptor-bound and free radioligand were separated by spin gel filtration assays as described previously [35]. Nonspecific binding was determined in the presence of 1 mM alprenolol. Radioactivity was counted on a MicroBeta TriLux scintillation counter (Perkin Elmer), and data were analyzed by nonlinear regression using Prism software (GraphPad).

For the thermostability determination of agonist-bound receptor in complex with the nanobody Nb80, stable mammalian cell lines were used expressing either wild type β_1_AR or β_1_AR-D87A^2.50^. Membranes were resuspended in Tm buffer (25 mM HEPES pH 7.5, 150 mM NaCl, 1 mM ascorbate, 0.1% BSA, 0.004% bacitracin and protease inhibitors) and homogenised with a 26-gauge needle. Nb80 and ^3^H-noradrenaline were then added to the membranes to final concentrations of 1 mg/mL and 200 nM respectively. Complex formation was allowed to occur on ice for 90 min, before the addition of DDM to a final concentration of 0.2%. Solubilisation was performed on ice for 1 h. Cell debris was removed by centrifugation for 5 min at 20,000 *g* and the supernatant was aliquoted into PCR strips prior to heating for 30 min at the indicated temperatures. The reaction was then quenched on ice for 30 min before loading 50 μL in duplicate on gel filtration columns. Receptor-bound and free radioligand were separated by gel filtration as described previously [35]. Nonspecific binding was determined in the presence of 2 mM noradrenaline hydrochloride. Radioactivity was counted on a MicroBeta TriLux scintillation counter (PerkinElmer), and data were analyzed by nonlinear regression using GraphPad Prism software. Results are the mean ± SEM for 2 independent experiments.

### Effect of Na^+^ ions on agonist affinity

High Five insect cells expressing the β_1_AR from recombinant baculovirus were lysed and nuclei were removed by centrifugation 5 min at 3000 *g*. Membranes were isolated by centrifugation (100,000 *g*, 30 min), washed 3 times with sodium-free buffer (Tris 20 mM and protease inhibitors), flash frozen in liquid nitrogen and stored at −80°C. The consecutive washes were equivalent to an effective dilution of 10^6^-fold, which is more than sufficient to reduce the Na^+^ ion concentration considerably below the K_D_ of Na^+^ binding to A_2A_R (40–50 mM). For competition binding assays, membranes were resuspended in 20 mM Tris pH 8 containing the desired concentration of NaCl or choline chloride (between 0 M and 1 M) and homogenised by 5 passages through a hypodermic syringe needle (26 G). Isoprenaline (0 mM to 1 mM final concentration) and ^3^H-DHA (10 nM final concentration) were added and the reactions incubated at room temperature for 2 h. Assays were terminated by filtration through 96-well GF/B filter plates pre-soaked with 0.1% polyethyleneimine and washed 3 times with the appropriate buffer. Plates were dried and radioactivity from bound ligand was counted on a liquid scintillation counter (Tri-Carb 2910 TR, Perkin Elmer). Data were analyzed by nonlinear regression using GraphPad Prism software.

### Nb80 efficacy in the presence or absence of NaCl

High Five insect cells expressing β_1_AR were resuspended in either buffer A (Tris 20 mM pH 8, 150 mM NaCl, 150 mM choline chloride, protease inhibitors) or buffer B (Tris 20 mM pH 8, 300 mM choline chloride, protease inhibitors) and lysed by 10 passages through a 26 G hypodermic syringe needle. The sample was then diluted and aliquoted and increasing concentrations (0 to 1.4 mg/mL) of purified Nb80 (in buffer A or B) were added. Isoprenaline was added (0 to 1 mM final concentration), incubated (1 h, 22°C), ^3^H-DHA added (final concentration of 20 nM) followed by a further incubation (1 h, 22°C). Receptor-bound radioligand was determined as above. The K_i_ for isoprenaline binding was determined and plotted versus each Nb80 concentration, and the EC_50_ of Nb80 was derived from a sigmoidal dose-response curve.

### Activity assays

Intracellular cAMP levels were measured using the cAMP-Glo Max kit (Promega). HEK293 cells stably expressing either wild type β_1_AR or β_1_AR-D87A^2.50^ were induced with 1 μg/mL doxycycline for 6 h. Cells were then detached in assay buffer (PBS, 500 μM IBMX, 100 μM Ro 20–1724, 30 mM MgCl_2_, 1 mM ascorbate) and counted. 5000 cells were treated with increasing concentrations of isoprenaline for 15 min at room temperature and cAMP assay was performed in a white, clear-bottom 96-well plate (Costar, 3610) according to the manufacturer's protocol. Luminescence was measured using the Pherastar plate reader. Titration of cAMP was performed to generate a standard curve and convert luminescence to levels of cAMP. Data were analyzed with GraphPad Prism software using a sigmoidal dose-response (variable slope) equation.

## Results

### Crystallisation and structure determination of β_1_AR-JM50

Structures of β_1_AR had previously been determined by crystallisation of a truncated thermostabilised mutant, β36-m23 [26]. Truncations were required to remove flexible regions at the N-terminus, the C-terminus and part of CL3, and mutations were required to improve thermostability (R68S, M90V, Y227A, A282L, F327A, F338M), remove the palmitoylation site (C358A) and to improve expression (C116L). The high thermostability of β36-m23 allowed its structure to be determined from crystals grown in octylthioglucoside [11] or Hega-10 [10], with complete data sets being collected from single crystals, but it formed only small crystals in LCP. In contrast, crystallisation of an ultra-thermostable mutant β_1_AR-JM50 [25] with cyanopindolol in LCP produced well-ordered plate-like crystals over 100 μm across. β_1_AR-JM50 is 12°C more thermostable than β36-m23 due to the inclusion of 3 additional thermostabilising mutations (I129V, D322K, Y343L). In this instance, it appears that there was a correlation between the improved thermostability of the receptor and improved size of the crystals produced in LCP.

The structure of β_1_AR-JM50 was determined to 2.1 Å resolution using diffraction data collected from a single cryo-cooled crystal (see Table 1 for crystallographic data). The overall structure is virtually identical to the 2.7 Å resolution structure of cyanopindolol-bound β36-m23 (PDB code 2VT4) [11] crystallised by vapour diffusion in the detergent octylthioglucoside (OTG), with an overall rmsd for all Cα atoms of 0.77 Å. The greatest variations between the receptors (up to a 2.6 Å shift) were observed at the extracellular surface of H1, H4 and extracellular loop 2 (EL2), and the intracellular surface composed of CL1, CL2, the intracellular ends of H5 and H6 and the linker between H7 and amphipathic H8 (Fig. S1 and Fig. S2). It is likely that the majority of these differences can be ascribed to differences in crystal contacts. There is also a small displacement of the extracellular end of H6-EL3-H7 towards the ligand binding pocket, which may be due partially to the engineered salt bridge (D322K–D200). Importantly the overall dimensions of the ligand binding pocket are identical between the structures of β_1_AR determined in LCP compared to OTG, with the distance between the Cαs of Ser211^5.42^ and Asn329^7.39^, which changes when either an agonist or antagonist is bound [36], being identical (16.0 Å) to within experimental error. There are many differences (above the noise level) in the rotamers of side chains between the two structures, although these mostly are oriented towards either the lipid bilayer or the regions outside the membrane. However, the one important exception is the rotamer of Ser212^5.43^. In previous structures of β_1_AR bound to antagonists, the hydroxyl side chain of Ser212^5.43^ participated in the formation of an intrahelical hydrogen bond with the backbone carbonyl of Ala208^5.39^ (ref [11]). In contrast, a hydrogen bond was observed between the side chains of Ser212^5.43^ and Asn310^6.55^ when agonists were bound [10]. In the cyanopindolol-bound β_1_AR LCP structure the side chains of Ser212^5.43^ and Asn310^6.55^ also form a hydrogen bond. The resolution of previous structures 2.3–2.7 Å resolution) was sufficient to define with reasonable certainty the rotamer for Ser212^5.43^, so the difference we see in the β_1_AR LCP structure is consistent with this hydrogen bond being dynamic when antagonists are bound to the receptor. In contrast, the agonist-bound structures of β_1_AR all contain the hydrogen bond between Ser212^5.43^ and Asn310^6.55^, suggesting it is important in receptor activation [10].

**Figure 1:**
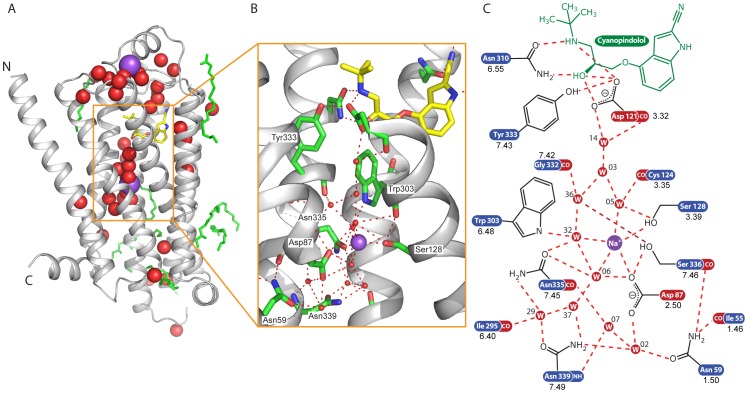
Structure of the intramembrane Na^+^ ion binding site. (*A*) Cartoon of β_1_AR (grey) depicting the positions of ordered water molecules (red spheres), Na^+^ ions (purple spheres), lipids (green sticks) and cyanopindolol (stick representation: carbon, yellow; nitrogen, blue; oxygen, red). The extracellular surface is at the top of the figure with the N-terminus (N) and C-terminus (C) labelled appropriately. (*B*) Detail of the Na^+^ binding site with portions of H2 and H3 removed for clarity: red spheres, water molecules; purple sphere, Na^+^ ion; green sticks, amino acid side chains; yellow sticks, cyanopindolol; red dashed lines, polar contacts, as defined by PyMOL. (*C*) Diagrammatic representation of the hydrogen bond network. Hydrogen bonds are assigned as displayed in COOT with a maximum distance of 3.5 Å. Where more than four interactions are shown, those with more favourable distances and geometry have been selected, with the exception of waters coordinating the Na^+^ ion, where up to 5 interactions are shown. Only the last two digits of the water numbers are shown.

The LCP structure of β_1_AR-JM50 allowed the identification of 38 water molecules, 7 lipid or detergent molecules and 2 bound Na^+^ ions per receptor. Interestingly, electron densities corresponding to cholesteryl hemisuccinate (CHS) were not observed despite it being present in the LCP crystallisation matrix, although ordered cholesterol or CHS molecules are observed in other structures of GPCRs where they often lie at the interface between crystallographic dimers (see for example [10], [37], [38]). The Na^+^ ion bound in EL2 was identified previously [11], but the intramembrane Na^+^ ion was not, due to its unexpected position within the hydrophobic core of the receptor (Fig. 1). Two lines of evidence suggest that the density at this position probably represents a Na^+^ ion rather than a water molecule. Firstly, the site is within 2.2–2.6 Å of 5 densities assigned to 3 water molecules, the hydroxyl oxygen of Ser128^3.39^ and a carboxyl oxygen of Asp87^2.50^. This results in the proposed Na^+^ ion being co-ordinated with 5 oxygen atoms in a distorted square pyramidal configuration (Fig S3), as observed in many other protein structures [39]. Secondly, the density aligns exactly with the intramembrane Na^+^ ion identified in the 1.8 Å resolution structure of the adenosine A_2A_ receptor (A_2A_R) [38], which is discussed further in the following section.

### Comparison of the Na^+^ ion binding site in β_1_AR and A_2A_R

**Figure 2:**
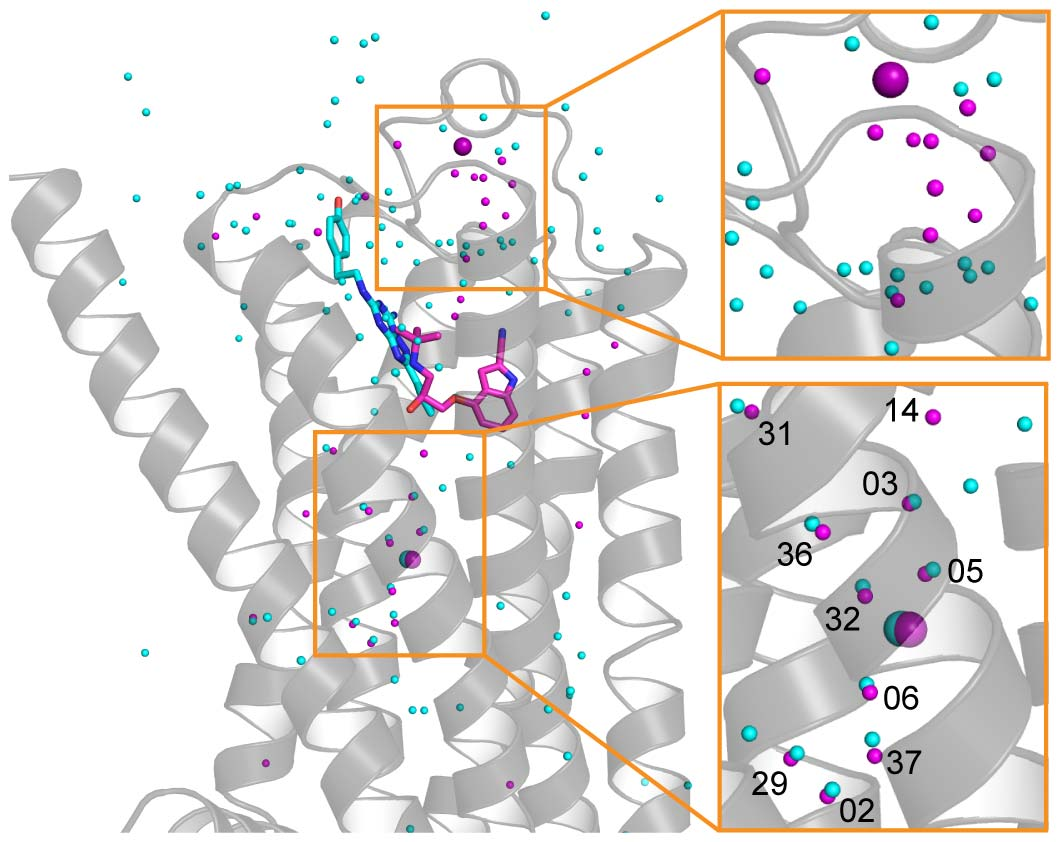
Conservation of the intramembrane Na^+^ ion and associated water network in β_1_AR and A_2A_R. An alignment between cyanopindolol-bound β_1_AR (PDB code 4BVN) and ZM241385-bound A_2A_R (PDB code 4EIY) [38] was performed and the positions of waters (small spheres), Na^+^ ions (large spheres) and ligands (sticks) compared superposed on the surface of representation of β_1_AR (grey): purple, β_1_AR; cyan, A_2A_R. The last two digits for the number of each water molecule in β_1_AR is shown in the bottom inset. The alignment was performed based on the polar side chains in the solvent channel that are conserved between β_1_AR and A_2A_R (β_1_AR/A_2A_R: N339/284^7.49^, N59/24^1.50^, D87/52^2.50^, S128/91^3.39^, N335/280^7.45^, Ser336/281^7.46^, W303/246^6.48^) with an overall RMSD of 0.30 for 30 atoms.

β_1_AR and A_2A_R both belong to the rhodopsin family (Class A) of GPCRs and share 23% identity in their amino acid sequences, excluding extensions of the N-terminus, C-terminus and CL3 found in β_1_AR but not in A_2A_R. Comparison of the 2.1 Å resolution structure of β_1_AR-JM50 and the 1.8 Å resolution structure [38] of A_2A_R-BRIL (excluding the BRIL portion of the fusion protein inserted into CL3) shows that the overall rmsd is 2.4 Å. In contrast, the Cα atoms of 7 polar amino acid residues conserved between β_1_AR and A_2A_R that co-ordinate either the Na^+^ ion or the associated water network, reveal considerable conservation of the structure of this region (rmsd 0.3 Å). These residues represent a proportion of conserved amino acid residues that were proposed to line the intramembrane Na^+^ ion binding pocket in many GPCRs (amino acid residues Leu^2.46^, Ala^2.49^, Asp^2.50^, Ser^3.39^, Trp^6.48^, Asn^7.45^, Asn^7.49^) and were identified by the alignment of amino acid sequences of Family A GPCRs [38]. Even more remarkable is the conservation in the positions of 8 water molecules in the associated water network (Fig. 2). In contrast, there is no such alignment in the positions of the water molecules in the extracellular portion of the receptors. This is expected because the ligand binding pockets, the positions of the ligands and the overall structure of the extracellular loops are not conserved between β_1_AR and A_2A_R.

### Effect of Na^+^ ion concentration on the pharmacology of β_1_AR

**Figure 3:**
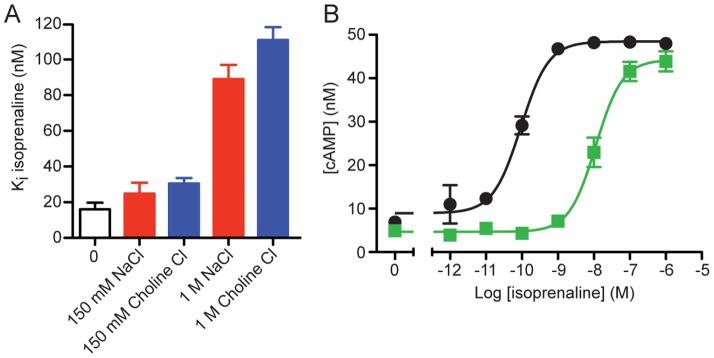
The effect of Na^+^ ion concentration on the binding of agonist to β_1_AR. (*A*) Competition binding assays were performed on β_1_AR in insect cell membranes using ^3^H-DHA and the agonist isoprenaline (Fig. S4) in the presence of either NaCl or choline chloride (0 mM, 150 mM, 1 M). IC_50_ values were converted to K_i_ values using the Cheng-Prusoff equation [66] using values of 4 nM for the K_D_ of ^3^H-DHA (0 mM, 150 mM or 1 M NaCl) and 10 nM for the concentration of ^3^H-DHA in the assay. Values (±SEM) were determined from 4 independent experiments performed in duplicate. (*B*) The activation of β_1_AR (black circles) and β_1_AR-D87A^2.50^ (green squares) by isoprenaline was monitored by measuring intracellular concentrations of cAMP in the cell lines 293-β_1_AR and 293-β_1_AR-D87A^2.50^. Expression levels of β_1_AR and β_1_AR-D87A^2.50^ were identical in both cell lines (Fig. S7). The EC_50_ for isoprenaline activation was 0.12±0.03 nM and 8.1±3.4 nM for β_1_AR and β_1_AR-D87A^2.50^, respectively. Results are the mean ± SEM for 4 independent experiments.

Identification of the intramembrane Na^+^ ion in the structure of A_2A_R was supported by pharmacological data indicating that Na^+^ acts as an allosteric antagonist [21], [38], [40], [41] *i.e.* the affinity for agonists decreased in the presence of increased concentrations of Na^+^. In contrast, there were no indications in the literature that Na^+^ ions had a similar effect on β_1_AR. However, data on β_2_AR suggested Na^+^ ions may be important for activity [42], particularly when considered in conjunction with data on the β_2_AR mutants D79A^2.50^ or D79N^2.50^ that showed decreased affinity of agonist binding and reduced receptor-mediated activation of G proteins [43], [44]. We therefore measured the affinities of agonist binding to membrane-bound wild-type β_1_AR in the presence of either 0 mM, 150 mM or 1 M NaCl. Competition binding assays using the agonist isoprenaline showed that there was no effect of Na^+^ ion concentration on the affinity of agonist binding compared to Na^+^-negative controls containing choline chloride of identical molarity (Fig. 3 and Fig. S4).

When agonists bind to β_1_AR and β_2_AR the overall structure remains in the R state [8], [10], with the R* state only being stabilised upon the binding of either a G protein [7] or a G protein mimetic such as the heavy chain camelid antibody fragment Nb80 [6]. A consequence of Nb80 binding to β_2_AR is a 100-fold shift in the affinity of agonist binding [6], so this effect can be used to study transitions between R and R*. Nb80 binds effectively to β_1_AR, so we studied the effect of Na^+^ ions on the efficacy of Nb80 to elicit a shift in agonist affinity in β_1_AR. The data showed that Nb80 was equally effective in increasing the affinity of agonist binding in both the presence and absence of Na^+^ ions, with the EC_50_ for Nb80 being 2.0 μM and 1.8 μM, respectively (Fig S5). Isothermal calorimetry (ITC) measurements of the affinity between β_1_AR and Nb80 showed no significant changes in affinity due to Na^+^ ion concentration (no Na^+^, K_D_ 350±80 nM; 150 mM Na^+^, K_D_ 740±230 nM; n = 5; p = 0.11) (Fig. S6). The agonist competition binding assays and the Nb80 efficacy experiments together suggest that the intramembrane Na^+^ ion is not involved in altering the equilibrium between the inactive state R and the activated state R* in β_1_AR.

A prediction from the experiments above is that if the Na^+^ ion binding site would be abolished by mutating Asp87^2.50^ to Ala, then in cell signalling assays there should not be an increase in basal activity of the receptor *i.e.* activity in the absence of agonist. This is at odds with what has been reported in the literature where D104A^2.50^ mutant in human β_1_AR was shown to have slightly increased basal activity [45]. We therefore constructed two stable cell lines in HEK293(TetR) cells expressing either wild-type β_1_AR or β_1_AR-D87A^2.50^ under the control of a tetracycline inducible promoter. This allows the tuning of expression levels of the receptor to give expression levels appropriate to the assay system used. Both cell lines expressed either β_1_AR or β_1_AR-D87A^2.50^ to similar levels (Fig. S7), which allowed the direct comparison of basal activity of the receptor constructs. The level of receptor activity in the absence of agonist was found to be slightly higher for the wild-type receptor, regardless of whether the expression levels of receptor were high or low (Fig. 3). In competition assays, isoprenaline bound to the D87A^2.50^ mutant as effectively as to the wild-type receptor (K_i_ values: β_1_AR, 0.9 μM; β_1_AR-D87A, 1.4 μM) (Fig. S7). Interestingly, the EC_50_ for isoprenaline to induce cAMP accumulation through receptor activation is raised markedly for β_1_AR-D87A^2.50^ (Fig. 3; β_1_AR, 0.12±0.03 nM; β_1_AR-D87A^2.50^, 8.1±3.4 nM; n = 4). Thus Asp87^2.50^ plays an important role in G protein activation, but not in modulating levels of basal activity.

### Thermostability of β_1_AR mutants in the intramembrane Na^+^ binding site

**Figure 4:**
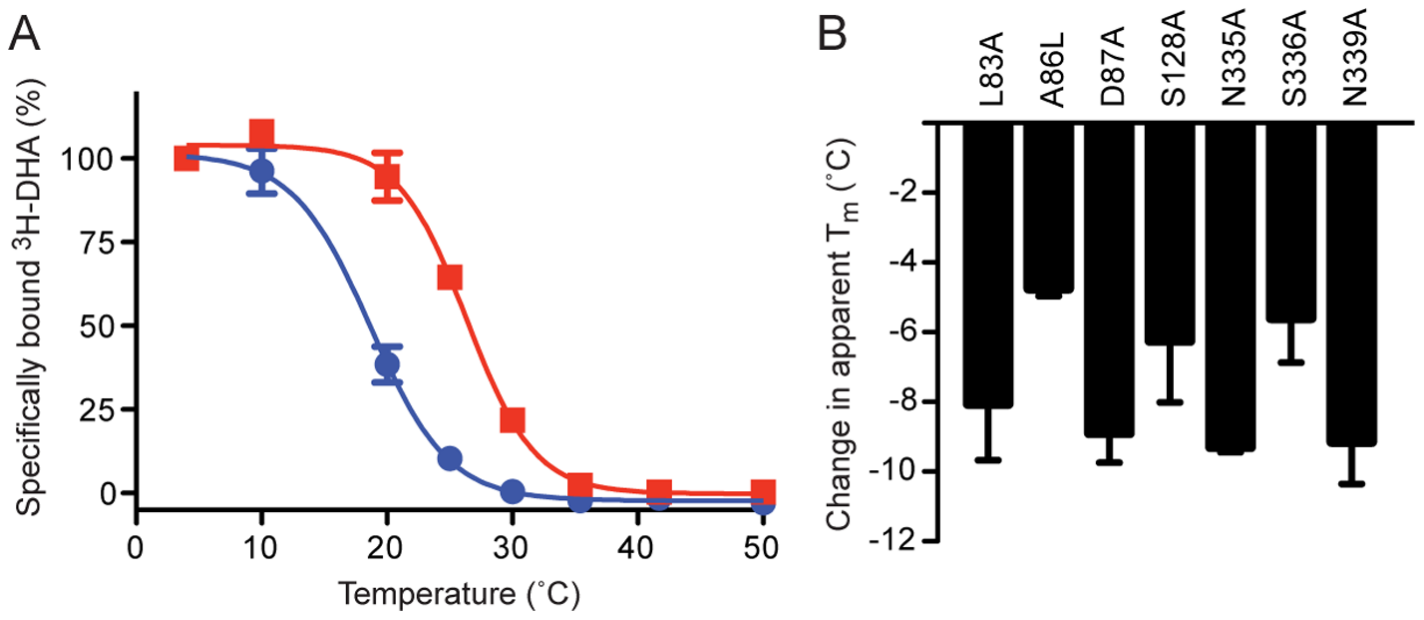
Thermostability of β_1_AR mutants. (*A*) The thermostability of dodecylmaltoside-solubilised β_1_AR was determined in either 150 mM NaCl (red squares) or 150 mM choline chloride (blue circles) giving apparent T_m_s (±SEM) of 26.6±0.3°C (n = 4) and 19.1±0.6°C respectively (n = 2). (*B*) The thermostability of detergent-solubilised β_1_AR mutants in the presence of 150 mM NaCl was determined (n = 2) and compared to the thermostability of wild-type β_1_AR. Mutations were made of all the residues lining the Na^+^ ion pocket and associated water network, but the mutations D121A, W303F and Y333A did not express any functional receptor as defined by ^3^H-DHA binding (Fig. S8).

The pharmacological data for β_1_AR measured in the presence or absence of Na^+^ ions are consistent in suggesting that Na^+^ ions do not play a direct role in modulating the R to R* transition. So why is there an intramembrane Na^+^ binding site in β_1_AR? If the role of Na^+^ is not pharmacological, then perhaps the role is structural. Measurement of the thermostability of ligand-free β_1_AR in the presence or absence of Na^+^ showed that the thermostability of β_1_AR dropped by 7.5°C in the absence of Na^+^ (Fig. 4), suggesting a direct role for intramembrane Na^+^ in maintaining the stability of the ligand-free receptor. In previous work we tested the thermostability of 318 Ala mutants throughout β_1_AR to identify those mutations that were thermostabilising, although we noted that many mutations were very destabilising [22]. Looking back through these data we noted that all the mutations in the intramembrane Na^+^ binding site appeared to be destabilising. As these assays were from single data points, the relevant mutants were expressed and the apparent T_m_ for each of the Ala mutants was determined (Fig. 4, Fig. S8). All 7 of the Ala mutants tested showed a decrease in thermostability compared to β_1_AR, with the largest effects being observed for D87A^2.50^, N335A^7.45^ and N339A^7.49^. In addition, we observed that the thermostability of noradrenaline-bound, Nb80-coupled β_1_AR-D87A^2.50^ was 9.4°C lower compared to β_1_AR, suggesting that Asp87^2.50^ also plays an important role in stabilising the R* state of β_1_AR (Fig. S9).

The discussion above focuses on the role of the intramembrane Na^+^ ion co-ordinated by Asp87^2.50^, but there is also another Na^+^ ion at the end of a short helix in extracellular loop 2 (EL2). The role of this Na^+^ on stabilising β_1_AR cannot be tested directly through mutagenesis, because the Na^+^ ion is co-ordinated by 3 backbone carbonyl groups and one or two water molecules. However, the decrease in thermostability of the β_1_AR-D87A^2.50^ mutant compared to β_1_AR is similar to the decrease in thermostability in β_1_AR in the absence of Na^+^. This suggests that the Na^+^ ion in EL2 does not play a significant role in stabilising β_1_AR.

## Discussion

The 2.1 Å resolution LCP structure of β_1_AR allows the comparison of virtually identical receptors bound to the same ligand crystallised in very different conditions using two different methodologies. In LCP crystallisation the receptor is in a bilayer composed of monoolein, whereas in vapour diffusion crystallisation the receptor is surrounded by detergent (octylthioglucoside). The overall structure is very similar and the ligand binding pocket is virtually identical. However, there are significant differences (up to 2.5 Å) in the loop regions of the receptor, probably due to the different packing interactions in the different crystals. This highlights the difficulty in assigning the role of loop regions in, for example, the activation of receptors where comparisons are made between receptors crystallised in different space groups [46]. In contrast, the high structural similarity in the transmembrane regions between the structures determined in detergent solution and in LCP lends confidence to the assumption that the crystal structures of receptors are good models for receptors, which is supported by the high predictive value of GPCR structures in structure-based drug design [47], [48].

The intramembrane Na^+^ ion binding site in Class A (rhodopsin family) GPCRs appears to be highly conserved as deduced from the conservation of amino acid residues involved in binding Na^+^ and the associated network of water molecules [38]. The structures of A_2A_R (1.8 Å resolution) [38], β_1_AR (2.1 Å resolution) and PAR1 (2.2 Å resolution) [49] all identify electron density in the structure that is consistent with a Na^+^ ion, based on the pattern of co-ordination and bond lengths. The Na^+^ ion is co-ordinated to a highly conserved residue, Asp^2.50^, which is conserved in 98% of GPCRs [50], and has been studied in a number of GPCRs by mutagenesis and functional assays. Mutation of Asp^2.50^ has a variety of effects on the GPCR including reduced or increased agonist affinity [43], [51]–[55], alteration of or no effect on G-protein coupling/signal transduction [53], [56]–[60] and loss of allosteric modulation by guanyl nucleotides or sodium [57], [61]. The fact that mutations at this site in different GPCRs results in a variety of different functional effects suggests that this residue is important in the equilibrium between R and R*. However, the precise effect is dependent on which particular receptor is being studied, which is probably due to variations between receptors in their energy landscapes and the kinetic barriers between different conformations. For turkey β_1_AR we did not observe any significant difference between the R-R* equilibrium either in the presence or absence of Na^+^ ions or in the mutant D87A^2.50^. Thus the activity of turkey β_1_AR is not modulated by Na^+^ ions.

The remarkable conservation of the Na^+^ binding site between A_2A_R and β_1_AR, including even the positions of co-ordinated water molecules, suggests that there was strong selective pressure for the maintenance of this structure during the evolution of GPCRs. *A priori*, the physiological role of a Na^+^ ion in modulating the activity of, for example A_2A_R, is unclear. Extracellular Na^+^ concentrations are tightly regulated in the body to 135–145 mM, and levels of 125 mM gives rise to severe hyponatremia [62]. Given that the EC_50_ for the allosteric affect of Na^+^ on A_2A_R is 40–48 mM [40], and also calculated from data in ref [21], it seems unlikely that the Na^+^ ion concentration is used physiologically to modulate the affinity of adenosine at A_2A_R under normal cellular conditions. Similarly, the EC_50_ for the allosteric affect of Na^+^ on the neurotensin receptor (NTSR1) is 43 mM [63]. Very short time scale fluctuations of Na^+^ do occur in the brain during synaptic transmission, but most hormonal control occurs on much longer timescales. The β_1_AR behaves differently from A_2A_R in that the Na^+^ ion concentration does not affect agonist binding. This difference probably arises due to the different effects of the agonists (without G proteins) on the conformational state of the receptors [36]. From the available crystal structures of β_1_AR and β_2_AR there are no significant differences between an antagonist-bound structure and a full agonist-bound structure except for a 1 Å contraction of the ligand binding pocket and rotamer changes of Ser^5.42^ and Ser^5.46^ (refs [8], [10], [13]). In contrast, the crystal structure of agonist-bound A_2A_R shows significant conformation changes so that the resulting structure is very similar to the R* state, even in the absence of a G protein [64], [65].

If the physiological role of the intramembrane Na^+^ ion is not to modulate the binding of agonists, then why is it there? One possibility is that the Na^+^-water network plays a role in stabilising the receptor in the ligand-free state. This is suggested from two observations. Firstly, the stability of the receptor decreases in the absence of Na^+^. Secondly, mutation of amino acid residues that form the intramembrane Na^+^-water binding pocket also decreases the thermostability of β_1_AR. Receptor stability was measured on detergent-solubilised mutants to get robust and rapid measurements, but the relative stability of the mutants in relation to one another is likely be the same in membranes, although the absolute values will obviously be different as the membrane is a more stabilising environment than detergent. Clearly there could be many different evolutionary strategies to receptor stabilisation that do not involve a Na^+^-water network, such as an intramembrane salt bridge, hydrogen bonds between side chains or extensive Van der Waals interactions between hydrophobic residues. Undoubtedly many of these alternatives could also be considerably more stable. However, consideration also has to be given to the transition between the R state and R* upon agonist binding, and also the stability of the R* state itself. Clearly, the energetics between R and R* need to be sufficiently low so that binding of a small molecule, such as adrenaline or noradrenaline, increases the probability of R* formation sufficiently for the receptor to function effectively. In addition, the R* state needs to be sufficiently stable to allow G protein activation. The only structure currently of a non-rhodopsin GPCR in the R* state is β_2_AR coupled to G protein [7], and in this structure the intramembrane Na^+^ ion and associated water network is displaced due to the movement of helices H2, H3 and H7 towards the core of the receptor, and Asp79^2.50^ forms hydrogen bonds to the side chains of Ser120^3.39^, Ser319^7.46^ and Asn321^7.49^. A similar arrangement of these conserved amino acid residues is seen in the active-like states of NTSR1 [63] and A_2A_R [64], [65], although in the latter case 2 water molecules are still co-ordinated to Asp52^2.50^ (ref [64]), supporting the contention that the agonist-bound A_2A_R structures may not be in the fully activated state [36].

The evolution of GPCRs has had to finely balance the stabilities of the R state and R* state, with a low energy of transition between them, so that they can function as efficient sensors of small molecules and activators of G proteins. The solution that has evolved balances these requirements by optimising the packing of amino acid side chains in the R* state and using mobile elements (Na^+^, water) to stabilise the structure in the absence of ligand. Thus the Na^+^ and water create a ‘soft’ interface between transmembrane helices H2, H3, H6 and H7 that is sufficient to stabilise the ligand-free structure, but is of sufficiently low energy to be easily disrupted on agonist binding to increase the probability of the R to R* transition. Given the high conservation of Asp^2.50^ and the side chains lining the intramembrane Na^+^ ion binding pocket, it is likely that the physiological role of the Na^+^ ion and its associated water network is similarly conserved in the function of many GPCRs.

### Accession numbers

The co-ordinates and structure factors for the structure of β_1_AR-JM50 bound to cyanopindolol have been deposited in the Protein Data Bank with accession number 4BVN.

## Supporting Information

Figure S1 Differences in structure between β_1_AR-JM50 crystallised in LCP and β_1_AR-m23 crystallised in octylthioglucoside.
(PDF)

Figure S2 Comparison of β_1_AR-JM50 and β_1_AR-m23 by B factor.
(PDF)

Figure S3 Coordination of the intra-membrane sodium ion in β_1_AR.
(PDF)

Figure S4 Agonist binding to β_1_AR was unaffected by the Na^+^ ion concentration in the presence or absence of Nb80.
(PDF)

Figure S5 The binding of the agonist isoprenaline to membrane-bound β_1_AR.
(PDF)

Figure S6 Nb80 affinity to the receptor was unaffected by the presence of sodium ions.
(PDF)

Figure S7 Similar expression levels and isoprenaline affinity of β_1_AR and β_1_AR-D87A^2.50^ expressed in tetracycline-inducible HEK293 stable cell lines.
(PDF)

Figure S8 Mutations lining the Na^+^-water binding pocket destabilise the ligand-free state of the receptor.
(PDF)

Figure S9 The D87A mutation destabilises the R* state of the receptor.
(PDF)
