# Supplementary material for: The 2.1 Å Resolution Structure of Cyanopindolol-Bound β1-Adrenoceptor Identifies an Intramembrane Na+ Ion that Stabilises the Ligand-Free Receptor
Source: PLoS One. 2014 Mar 24;9(3):e92727. doi: 10.1371/journal.pone.0092727 (PMC3963952; doi:10.1371/journal.pone.0092727)
Supplement: Figure S1 — Differences in structure between β1AR-JM50 crystallised in LCP and β1AR-m23 crystallised in octylthioglucoside. (PDF) [file pone.0092727.s001.pdf]

A

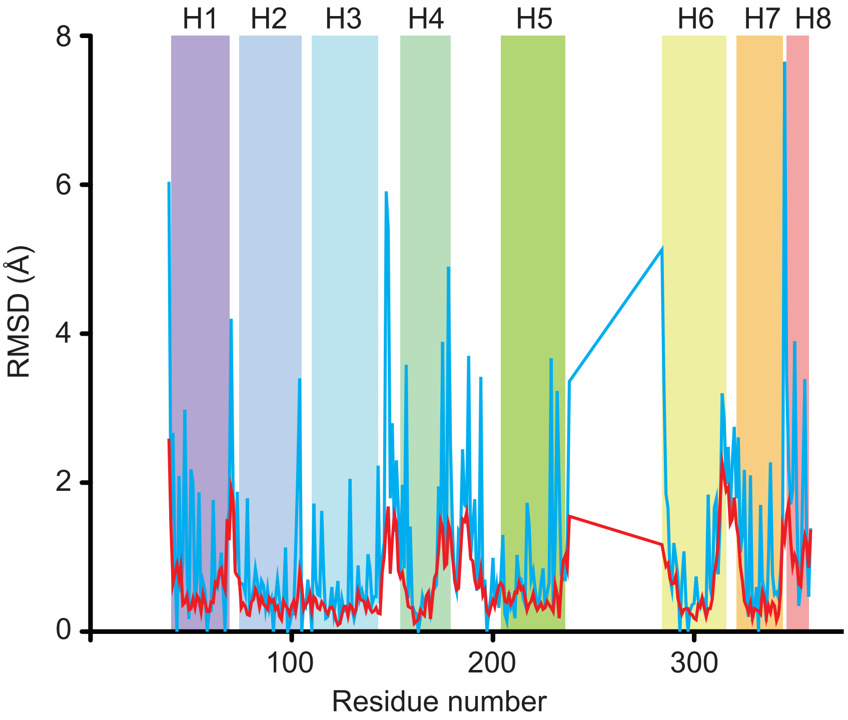

B

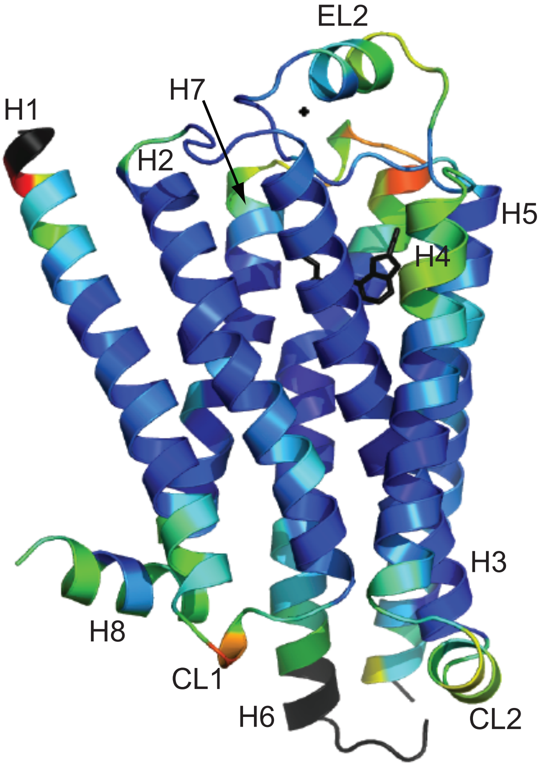

Fig. S1. Differences in structure between β_1AR-JM50 crystallised in LCP and β_1AR-m23 crystallised in octylthioglucoside. (A) RMSD of the main chain (red) and side chains (blue) of each residue of β_1AR-JM50 aligned to β_1AR-m23 (PDB code 2VT4, chain B) with LSQKAB (Kabsch, 1976). Transmembrane regions are depicted by coloured bars and labeled H1-H8. (B) Displacement of Cα atoms of β_1AR-JM50 from the β_1AR-m23 structure are shown in rainbow colouration; blue, low displacement; red, high displacement. RMSD of Cα atoms is 0.61 Å (TM helices) and 0.77 Å (all residues).
