# Supplementary material for: The 2.1 Å Resolution Structure of Cyanopindolol-Bound β1-Adrenoceptor Identifies an Intramembrane Na+ Ion that Stabilises the Ligand-Free Receptor
Source: PLoS One. 2014 Mar 24;9(3):e92727. doi: 10.1371/journal.pone.0092727 (PMC3963952; doi:10.1371/journal.pone.0092727)
Supplement: Figure S2 — Comparison of β1AR-JM50 and β1AR-m23 by B factor. (PDF) [file pone.0092727.s002.pdf]

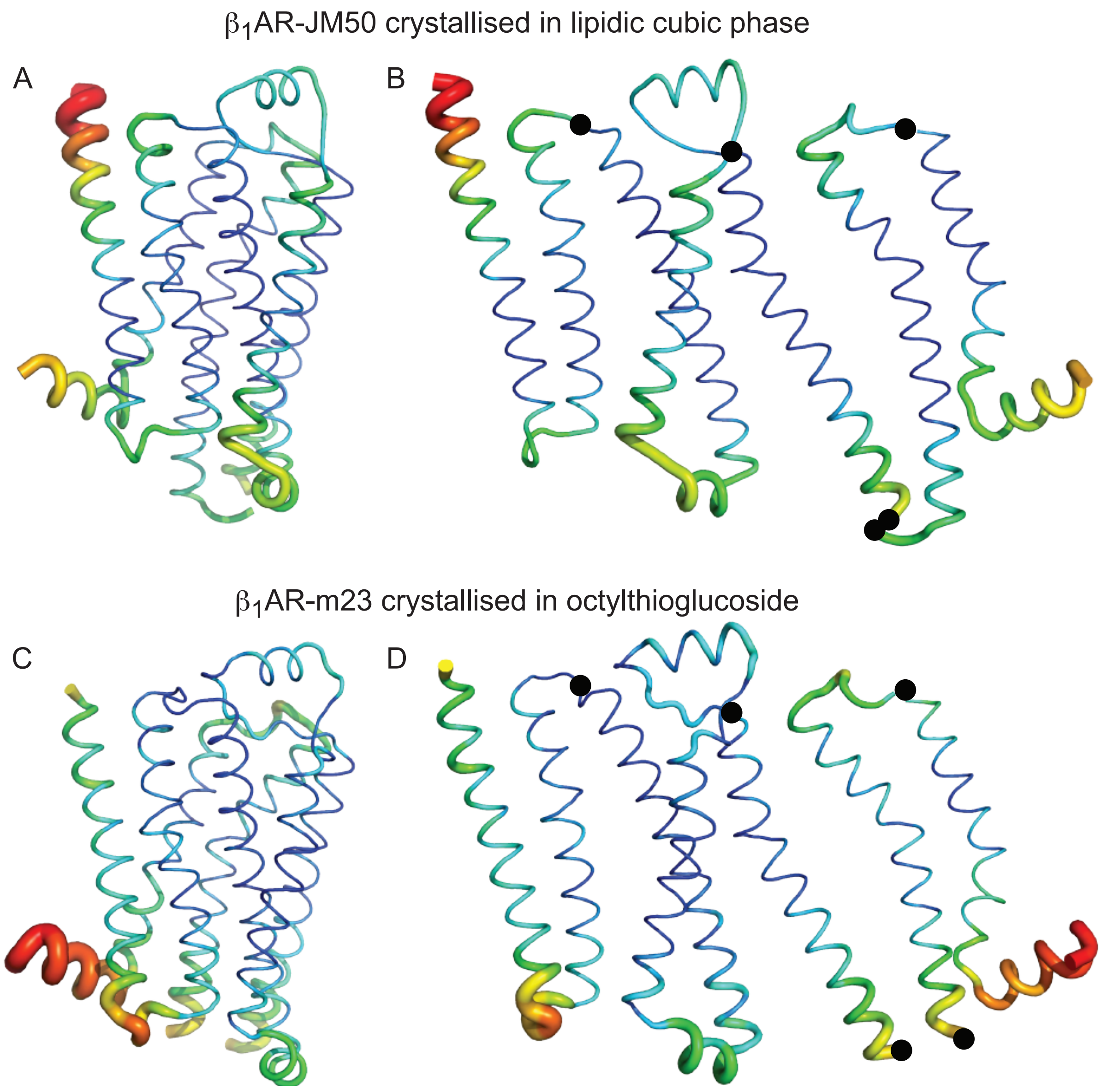

Fig. S2. Comparison of β_1AR-JM50 and β_1AR-m23 by B factor. B factors are indicated by the thickness of the ribbon and the rainbow colouration (thin & blue, low B factor; thick & red, high B factor); (A, B) β_1AR-JM50, (C, D) β_1AR-m23. Panels B and D depict the receptor in extended conformation where the black dots show the pivot points about which regions of the receptor were rotated.
