# Supplementary material for: The 2.1 Å Resolution Structure of Cyanopindolol-Bound β1-Adrenoceptor Identifies an Intramembrane Na+ Ion that Stabilises the Ligand-Free Receptor
Source: PLoS One. 2014 Mar 24;9(3):e92727. doi: 10.1371/journal.pone.0092727 (PMC3963952; doi:10.1371/journal.pone.0092727)
Supplement: Figure S3 — Coordination of the intra-membrane sodium ion in β1AR. (PDF) [file pone.0092727.s003.pdf]

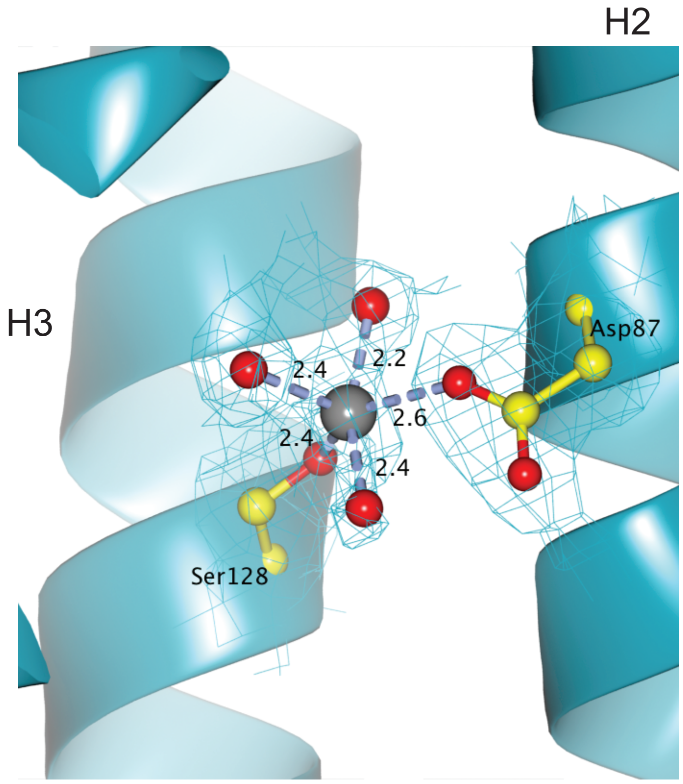

Fig S3. Coordination of the intra-membrane sodium ion in β1-AR
The sodium ion (gray sphere) is coordinated by 3 water molecules (red spheres), the γ–hydroxyl of Ser128 and a carboxylate oxygen of Asp87. Helices H2 and H3 are shown in ribbon representation. 2Fo-Fc density (sharpened by a B factor of -20 and contoured at 1 Å) is shown for the two side chains, the sodium ion and the coordinating waters. Sodium coordination distances are in Å.
