# Supplementary material for: The 2.1 Å Resolution Structure of Cyanopindolol-Bound β1-Adrenoceptor Identifies an Intramembrane Na+ Ion that Stabilises the Ligand-Free Receptor
Source: PLoS One. 2014 Mar 24;9(3):e92727. doi: 10.1371/journal.pone.0092727 (PMC3963952; doi:10.1371/journal.pone.0092727)
Supplement: Figure S4 — Agonist binding to β1AR was unaffected by the Na+ ion concentration in the presence or absence of Nb80. (PDF) [file pone.0092727.s004.pdf]

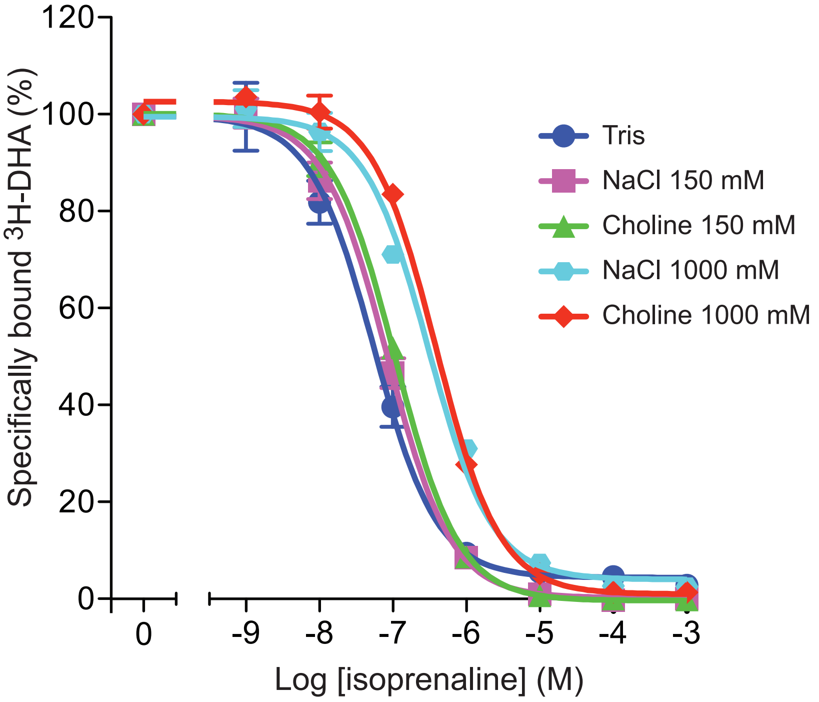

Fig. S4. Agonist binding to β_1AR was unaffected by the Na^+ ion concentration. Competition binding assays were performed on wild type β_1AR in insect cell membranes using ^{3}H-DHA and the agonist isoprenaline in different solution conditions (n = 4 of duplicate measurements). K_i values (mean ± SEM) were as follows: 0 mM (blue circles), K_i = 16.1 ± 3.7 nM; 150 mM NaCl (purple squares), K_i = 24.8 ±6.1 nM; 150 mM choline chloride (green triangles), K_i = 30.6 ± 2.9 nM; 1 M NaCl (cyan hexagons), K_i = 89.1 ± 8.0 nM; 1 M choline chloride (red diamonds), K_i = 111 ± 7.0 nM.
The data are plotted as histograms in Figure 3.
