# Supplementary material for: The 2.1 Å Resolution Structure of Cyanopindolol-Bound β1-Adrenoceptor Identifies an Intramembrane Na+ Ion that Stabilises the Ligand-Free Receptor
Source: PLoS One. 2014 Mar 24;9(3):e92727. doi: 10.1371/journal.pone.0092727 (PMC3963952; doi:10.1371/journal.pone.0092727)
Supplement: Figure S5 — The binding of the agonist isoprenaline to membrane-bound β1AR. (PDF) [file pone.0092727.s005.pdf]

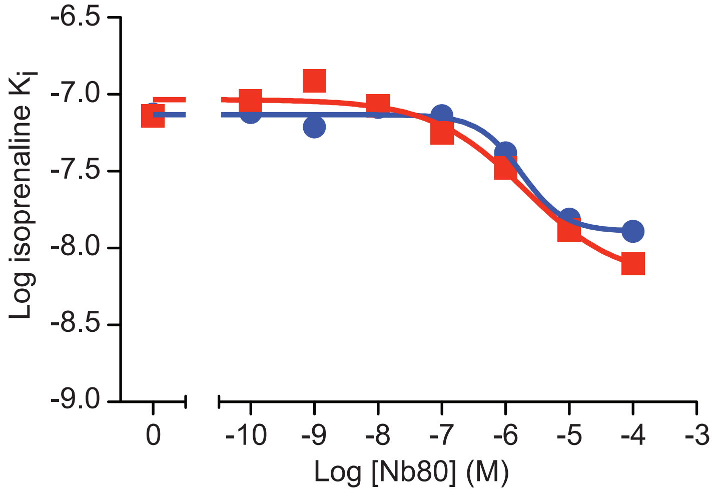

Fig. S5. The binding of the agonist isoprenaline to membrane-bound β_1AR was determined from competition binding curves using ^{3}H-DHA under conditions of increasing concentrations of the nanobody Nb80 and in the absence (blue circles) or presence (red squares) of 150 mM NaCl. IC_{50} values were determined from a single experiment performed in duplicate and converted to K_i values as above. The EC_{50} for Nb80 was 1.77 µM in the presence of 150 mM choline chloride and 2.0 µM in the presence of 150 mM NaCl.
