# Supplementary material for: The 2.1 Å Resolution Structure of Cyanopindolol-Bound β1-Adrenoceptor Identifies an Intramembrane Na+ Ion that Stabilises the Ligand-Free Receptor
Source: PLoS One. 2014 Mar 24;9(3):e92727. doi: 10.1371/journal.pone.0092727 (PMC3963952; doi:10.1371/journal.pone.0092727)
Supplement: Figure S6 — Nb80 affinity to the receptor was unaffected by the presence of sodium ions. (PDF) [file pone.0092727.s006.pdf]

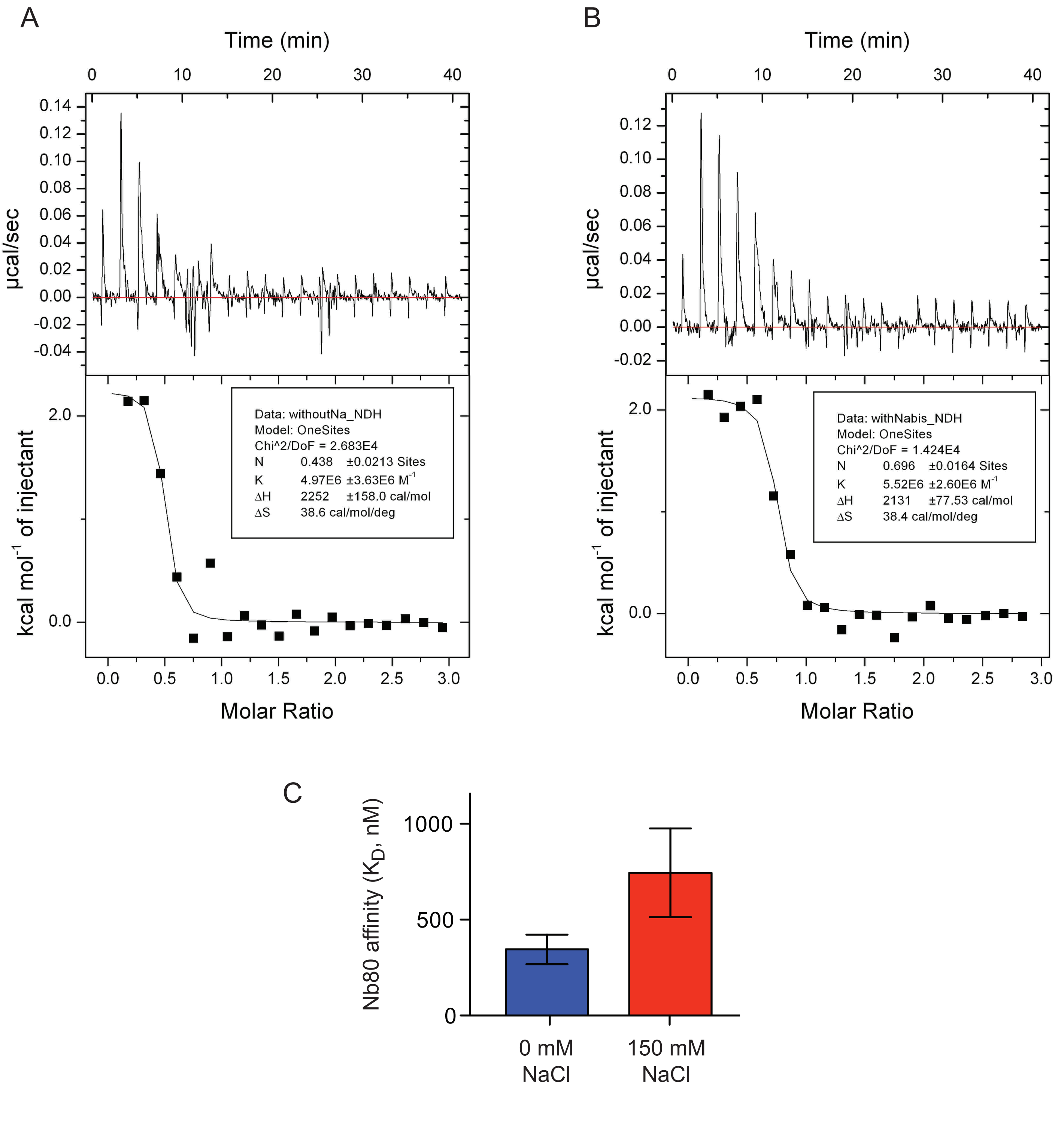

Fig. S6. Nb80 affinity to the receptor was unaffected by the presence of sodium ions. ITC measurements were performed on wild type β_1AR in the absence (A) or presence (B) of 150 mM NaCl. Association constants (K) were derived from the sigmoidal curve that was analysed using a single-binding-site model. K_D (=1/K) values were the mean ± SEM of 5 measurements: no NaCl, 350 ± 80 nM; 150 mM NaCl 740 ± 230 nM and are plotted as histograms (C). The Student t-test indicated that these values were not statistically different ($p = 0.11$).
