# Supplementary material for: The 2.1 Å Resolution Structure of Cyanopindolol-Bound β1-Adrenoceptor Identifies an Intramembrane Na+ Ion that Stabilises the Ligand-Free Receptor
Source: PLoS One. 2014 Mar 24;9(3):e92727. doi: 10.1371/journal.pone.0092727 (PMC3963952; doi:10.1371/journal.pone.0092727)
Supplement: Figure S7 — Similar expression levels and isoprenaline affinity of β1AR and β1AR-D87A2.50 expressed in tetracycline-inducible HEK293 stable cell lines. (PDF) [file pone.0092727.s007.pdf]

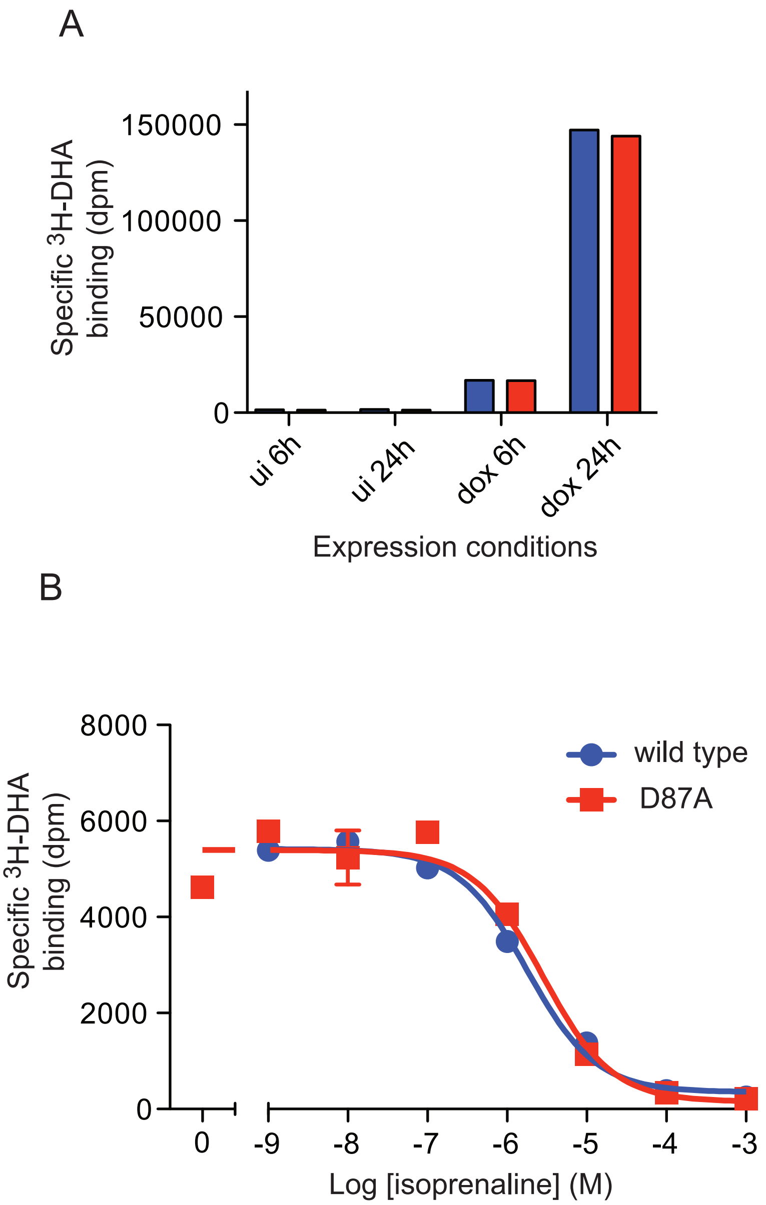


**Fig. S7.** Similar expression levels and isoprenaline affinity of $\beta_1$AR and $\beta_1$AR-D87A$^{2.50}$ expressed in tetracycline-inducible HEK 293 stable cell lines. (A) The Flp-in T-Rex system (Invitrogen) was used to generate stable cell lines expressing either wild type $\beta_1$AR (blue) or $\beta_1$AR-D87A$^{2.50}$ (red). Cells were either uninduced (ui) or induced with doxycycline (dox, 1 µg/mL) for the indicated time. Binding assays were performed on whole cells at a single saturating concentration of $^3$H-DHA binding (100 nM final concentration, 25x $K_D$). (B) Competition binding assays were performed on membranes from HEK293 cell lines stably expressing either wild type $\beta_1$AR (blue circles) or $\beta_1$AR-D87A$^{2.50}$ (red squares) using $^3$H-DHA and the agonist isoprenaline. $IC_{50}$ values were converted to $K_i$ values using the Cheng-Prusoff equation and values of 4 nM for the $K_D$ of $^3$H-DHA and 10 nM for the concentration of $^3$H-DHA in the assay: $K_i$ values: $\beta_1$AR, 0.9 µM; $\beta_1$AR-D87A$^{2.50}$, 1.4 µM.
