# Supplementary material for: The 2.1 Å Resolution Structure of Cyanopindolol-Bound β1-Adrenoceptor Identifies an Intramembrane Na+ Ion that Stabilises the Ligand-Free Receptor
Source: PLoS One. 2014 Mar 24;9(3):e92727. doi: 10.1371/journal.pone.0092727 (PMC3963952; doi:10.1371/journal.pone.0092727)
Supplement: Figure S8 — Mutations lining the Na+-water binding pocket destabilise the ligand-free state of the receptor. (PDF) [file pone.0092727.s008.pdf]

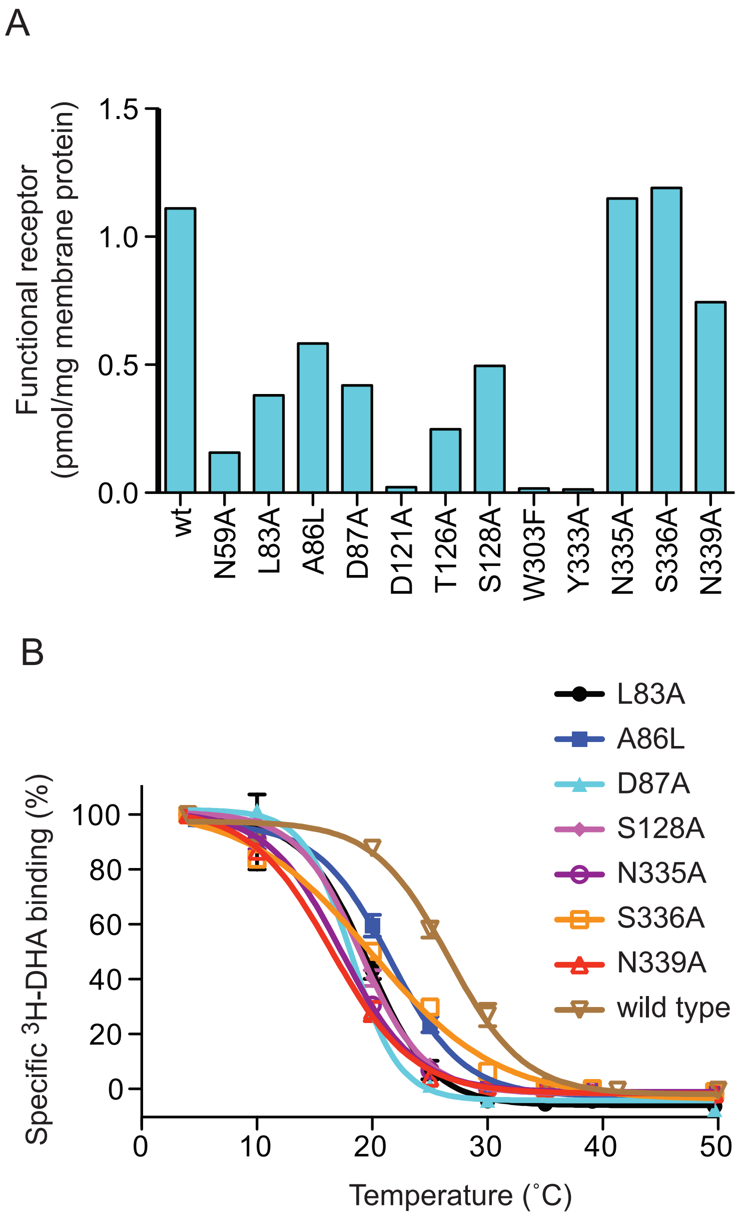

Fig. S8. Mutations lining the Na^+-water binding pocket destabilise the ligand-free state of the receptor. (A) Mutations were made of all the residues lining the Na^+ ion pocket and associated water network and expressed in E. coli. ^{3}H-DHA binding was performed at a single concentration (100 nM) on membranes to quantify the number of functional receptors, but the mutations D121A, W303F and Y333A showed no binding. (B) Thermostability assays were performed on the wild type and Ala/Leu mutants. Detergent-solubilised receptor was heated in the ligand-free state for 30 min at the indicated temperatures before adding ^{3}H-DHA. Apparent T_m values from 2 independent experiments were determined: wild type β_1AR, 26.6 ± 0.25 °C; L83A, 18.4 ± 1.1 °C; A86L, 21.7 ± 0.1 °C; D87A, 17.6 ± 0.6 °C; S128A, 20.2 ± 1.2 °C; N335A, 17.2 ± 0.1 °C; S336A, 20.9 ± 0.9 °C; N339A, 17.3 ± 0.8 °C. The T_m difference between the mutants and wild type (ΔT_m) is shown in Figure 4.
