# Supplementary material for: The 2.1 Å Resolution Structure of Cyanopindolol-Bound β1-Adrenoceptor Identifies an Intramembrane Na+ Ion that Stabilises the Ligand-Free Receptor
Source: PLoS One. 2014 Mar 24;9(3):e92727. doi: 10.1371/journal.pone.0092727 (PMC3963952; doi:10.1371/journal.pone.0092727)
Supplement: Figure S9 — The D87A mutation destabilises the R* state of the receptor. (PDF) [file pone.0092727.s009.pdf]

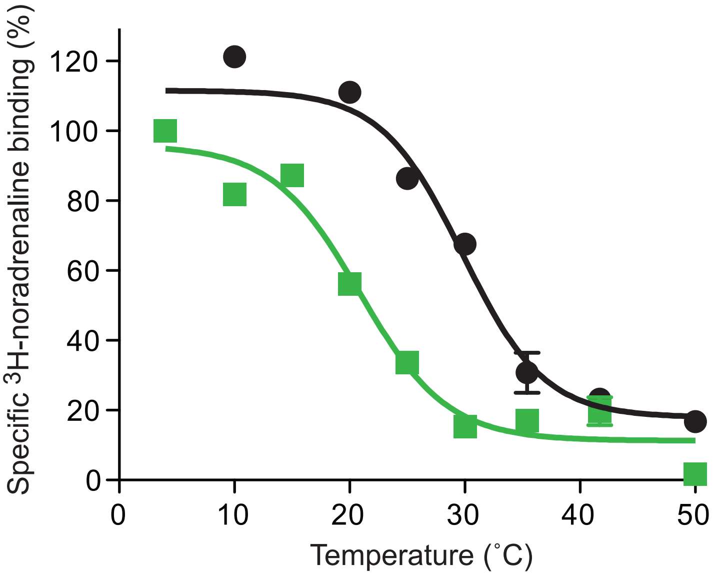

Fig. S9. The D87A mutation destabilises the R* state of the receptor. The thermostability of detergent-solubilised β_1AR (black circles) and β_1AR-D87A$^{2.50}$ (green squares) was determined in the R* state in the presence of 1 mg/mL Nb80 and 200 nM of ^{3}H-noradrenaline giving apparent T_ms (± SEM) of 29.1 ± 0.7 °C and 19.7 ± 1.2 °C respectively (n = 2).
